# 表面改性微芯片电泳分离分析保健品中的功效成分

**DOI:** 10.3724/SP.J.1123.2023.08019

**Published:** 2023-10-08

**Authors:** Waichun LAU, Yali CHEN, Ling XIA, Xiaohua XIAO, Gongke LI

**Affiliations:** 中山大学化学学院, 广东 广州 510006; School of Chemistry, Sun Yat-sen University, Guangzhou 510006, China

**Keywords:** 表面改性, 微芯片, 电泳, 环烯烃共聚物, 保健品, surface modification, microchips, electrophoresis, cycloolefin copolymer (COC), health care products

## Abstract

微芯片电泳(microchip electrophoresis, MCE)分离效率高、试剂和样品消耗量少、易实现多操作单元集成,在食品、环境和药物等领域应用广泛。环烯烃共聚物(cycloolefin copolymer, COC)等聚合物微芯片材料成本低、制作简便,但电泳过程中通道表面易发生非特异性吸附,且电渗流不稳定,限制了其应用。这些不足可通过COC表面改性解决。本文采用静态涂层和动/静态涂层联合策略,研制通道表面改性COC微芯片,结合激光诱导荧光(laser-induced fluorescence, LIF)检测,发展了简单高效的MCE-LIF分离分析方法,用于保健品中功效成分检测。通过负电荷涂层微通道表面改性方法,可以提高MCE对阳离子氨基酸的分离效果,而通过动/静态涂层结合的微通道表面改性方法,可以提高MCE对阴离子氨基酸的分离效果。负电荷涂层由COC微通道表面的疏水氨基酸吸附、戊二醛固定化和亲水氨基酸功能化构建,正电荷涂层由COC微通道表面的缬氨酸吸附、羧基活化和乙二胺功能化构建,动态涂层通过向微通道引入含有羟丙基甲基纤维素与十二烷基硫酸钠的缓冲液自动形成。实验研究了表面改性微通道的理化性质和电泳分离的影响因素,并将所建立的方法用于儿童保健品中赖氨酸和*γ*-氨基丁酸以及运动饮料中天冬氨酸与牛磺酸MCE-LIF的检测,儿童保健品中赖氨酸和*γ*-氨基丁酸的加标回收率为84.8%~118%,相对标准偏差(RSD)≤7.2%,运动饮料中天冬氨酸与牛磺酸的加标回收率为97.5%~118%, RSD≤6.4%。分析结果与HPLC方法的测定结果吻合,该法在保健品中氨基酸分离分析中有应用前景。

## Abstract (en)

Microchip electrophoresis (MCE) is widely applied in food, environment, medicine, and other fields, owing to its high separation efficiency, low consumption of reagents and samples, and ease of integrating multiple operating units. Polymer microchip materials like cycloolefin copolymer (COC) are low-cost and easy to fabricate. However, their practical applications are limited by the non-specific adsorption on channel surface during electrophoresis and the instability of electroosmotic flow. These shortcomings can be solved by COC surface modification. In this study, a static coating and dynamic/static coating combined strategy was used to develop a channel-surface-modified COC microchip. Combined with laser-induced fluorescence (LIF) detection, a MCE-LIF separation and analysis method was developed for detecting functional components in health care products. The separation performance of MCE was improved by the static coating microchannel surface modification method. The static coating was constructed by hydrophobic amino acid adsorption, glutaraldehyde immobilization, and hydrophilic amino acid functionalization on the COC microchannel surface. The separation performance of MCE was improved by microchannel surface modification combined with dynamic/static coating. The static coating was constructed by valine adsorption, carboxyl activation, and ethylenediamine functionalization on the COC microchannel surface. The dynamic coating is automatically formed by introducing a buffer solution containing hydroxypropyl methylcellulose and sodium dodecyl sulfate into the microchannel. The physical and chemical properties of surface-modified microchannels and the factors governing electrophoretic separation were studied. Combined with LIF detection, the MCE-LIF separation and analysis of lysine and *γ*-aminobutyric acid present in children’s health care products, as well as aspartic acid and taurine in sport drinks, were developed. The recoveries of lysine and *γ*-aminobutyric acid in children’s health care products were 84.8%-118%, and the relative standard deviations (RSDs) were less than 7.2% (*n*=3). The recoveries of aspartic acid and taurine in sport drinks were 97.5%-118%, and the RSDs were less than 6.4% (*n*=3). The analysis results are consistent with the HPLC results, and the method has potential for application in the separation and analysis of anionic amino acids in health care products.

微芯片电泳(microchip electrophoresis, MCE)的分析效率比普通毛细管电泳(capillary electrophoresis, CE)高,且MCE系统试剂和样品消耗量少、易于高度集成、有助于现场分析,在生物医学和药物分析^[1]^、环境监测^[2]^、法医调查^[3]^和临床诊断^[4]^等方面应用广泛。

微芯片表面改性方法包括静态涂层改性和动态涂层改性。动态涂层通过在缓冲液中添加聚合物或表面活性剂改变电渗流(electroosmotic flow, EOF)来实现,操作方便且过程可逆,但涂层容易脱落。静态涂层改性是电泳微芯片最有效的表面改性方法,涂层稳定且可提高电泳重现性。常见静态涂层改性方法包括紫外接枝法^[5]^、等离子处理法^[6]^和多层化学沉积法^[7]^。采用阴离子静态涂层^[8]^对微通道进行改性,能产生阳离子EOF,用于阳离子分析物分离。保健品中常见的阳离子氨基酸有*γ*-氨基丁酸和赖氨酸。*γ*-氨基丁酸是哺乳动物中枢神经系统中主要的抑制性神经递质,它影响大脑发育,在促进神经元发育、预防失眠等方面发挥重要作用^[9]^。口服赖氨酸能增加生长激素释放,有助于人体钙吸收和肾脏保护,增加骨胶原交联过程^[10]^。阴离子静态涂层可提高MCE对阳离子氨基酸的分离效果,但不适用阴离子氨基酸分析。采用动/静态涂层结合的环烯烃共聚物(cycloolefin copolymer, COC)微通道表面改性方法,可提高MCE对阴离子氨基酸的分离效果。牛磺酸和天冬氨酸等阴离子氨基酸常作为有消除疲劳、提高记忆力等功效的保健品中的功效成分。牛磺酸是重要神经递质、抗氧化剂,能调节葡萄糖和脂质,影响能量代谢^[11]^,常被添加到婴儿食品和运动饮料中,是合法的食品添加剂^[12]^。天冬氨酸是有酸性侧链的蛋白源性氨基酸,能刺激神经元受体,是哺乳动物大脑中主要兴奋性神经递质^[13]^,它参与葡萄糖生成,可维持正常能量代谢,对瞬时脑力劳动负荷后能量修复有积极作用^[14]^。*γ*-氨基丁酸和赖氨酸常用检测方式包括高效液相色谱法(HPLC)^[15]^、比色法^[16]^、荧光传感器检测^[17]^、电化学检测^[18]^、CE^[19]^等。而牛磺酸和天冬氨酸检测方法有色谱法^[20]^、CE^[21]^、电化学法^[22]^、核磁共振法^[23]^等。

本研究采用光学性能好、惰性且低成本的COC作为MCE芯片基底材料,在COC微芯片通道表面通过疏水氨基酸吸附、戊二醛(glutaraldehyde, GA)固定化和亲水氨基酸功能化构建静态涂层,提高阳离子分析物的MCE分离效果,结合激光诱导荧光(laser-induced fluorescence, LIF)检测,用于儿童保健品中赖氨酸与*γ*-氨基丁酸MCE分离分析;采用动/静态涂层结合微芯片通道表面改性方法,通过缬氨酸吸附、羧基活化和乙二胺(ethylenediamine, EDA)功能化在COC微通道构建静态涂层,向微通道引入含有羟丙基甲基纤维素(hydroxypropyl methyl cellulose, HPMC)与十二烷基硫酸钠(sodium lauryl sulfate, SDS)的缓冲液形成动态涂层,提高阴离子分析物MCE分离性能,结合LIF检测,用于运动饮料中天冬氨酸与牛磺酸的分离分析。

## 1 实验部分

### 1.1 仪器与试剂

HVS448高压电源(美国LabSmith公司); DS-Ri2 CCD摄像机和ECLIPSE Ti2-U倒置荧光显微镜(日本尼康株式会社); FA 1604电子天平(上海天平仪器厂); CNC数控雕刻机(深圳市捷丰泰科技有限公司);自制LIF检测器(由455 nm激光二极管、505 nm DM505二色镜、520 nm BP520长通滤波器和AD500-8-TO52S2雪崩光电二极管构成); USB-6002多功能数据采集卡(National Instruments, USA); LC-2010C HT高效液相色谱仪(日本岛津株式会社); TGL-20M高速冷冻离心机(湖南湘仪实验室仪器开发有限公司); ESCALab250 X-射线光电子能谱仪(XPS,赛默飞,美国); DSA100接触角测量仪(克吕士,德国); JSM-6330F冷场发射扫描电镜(field emission scanning electron microscope, SEM,日本电子株式会社,日本)

丙氨酸(alanine, Ala, 99%(纯度,下同))、缬氨酸(valine, Val, 99%)、亮氨酸(leucine, Leu, 99%)、异亮氨酸(isoleucine, Ile, 98%)、甲硫氨酸(methionine, Met, 99%)、脯氨酸(proline, Pro, 99%)、色氨酸(tryptophan, Try, 99%)、苯丙氨酸(phenylalanine, Phe, 99%)、天冬氨酸(aspartic acid, Asp, 98%)、*γ*-氨基丁酸(*γ*-aminobutyric acid, GABA, 98%)、赖氨酸(lysine, Lys, 98%)、精氨酸(arginine, Arg, 99%)和牛磺酸(taurine, Tau, 98%)购于百灵威科技有限公司(北京)。1-乙基-(3-二甲基氨基丙基)碳二亚胺盐酸盐(1-(3-dimethylaminopropyl)-3-ethylcarbodiimide hydrochloride, EDC, 98%)、*N*-羟基琥珀酰亚胺(*N*-hydroxy succinimide, NHS, 98%)、硼砂(99%)和SDS(≥98.5%)、磷酸二氢钠(99.9%)、氢氧化钠(97%)与乙腈(色谱级)购于阿拉丁试剂有限公司(上海)。4-氟-7-硝基-2,1,3-苯并恶二唑(4-fluoro-7-nitro-2,1,3-benzoxadiazole, NBD-F, 99%)、HPMC(USP2910, 2%)购于上海麦克林生化有限公司(上海)。GA(50%)购于BBI生命科学有限公司(上海)。磷酸盐缓冲液(PBS, pH 7.2~7.6 (1×))购于赛国生物科技有限公司(广州)。碳酸氢钠(99%)、EDA(99%)、三水合乙酸钠和甲醇(色谱级)购于广州化学试剂厂,实验用水均为由Millipore纯化系统得到的超纯水(18.25 MΩ·cm)。C_18_色谱柱(250 mm×4.6 mm, 5 μm,北京DiKMA科技有限公司)。儿童保健品(压片糖果)和运动饮料购于零售店。

### 1.2 微芯片制作

COC微芯片通过数控铣床机械雕刻法建立微通道,使用打孔器打孔,通过热压技术封装构建,详细制作过程参考文献[24]。微芯片通道网络设计如图1a,包括4个通道段,所有通道均宽100 μm,深70 μm;分离通道长2.7 cm,其他3个通道长0.8 cm。微通道雕刻完成后在4个通道端处打孔,乙醇超声洗净后于40 ℃烘干。然后将其置于135 ℃烘箱中热压10 min封装构建微芯片。将5 mL离心管盖打孔后作为储液池,使用由COC溶解到甲苯中制作成的胶水将储液池连接到4个孔处,放置过夜。

### 1.3 表面改性和表征

#### 1.3.1 负电荷涂层

COC微通道表面负电荷涂层通过疏水氨基酸吸附、GA固定化、亲水氨基酸功能化构建,如图1b。首先,将2.0%(质量分数)疏水氨基酸/PBS溶液充满通道,室温静置30 min后用蒸馏水冲洗所有通道,38 ℃烘干;其次,将2.25%(v/v)GA水溶液充满通道,室温静置60 min后用蒸馏水冲洗所有通道,38 ℃烘干;最后,将2.5%(质量分数)亲水氨基酸/PBS溶液充满通道,室温静置30 min后用蒸馏水冲洗所有通道,38 ℃烘干制得微芯片,4 ℃保存备用。

**图 1:**
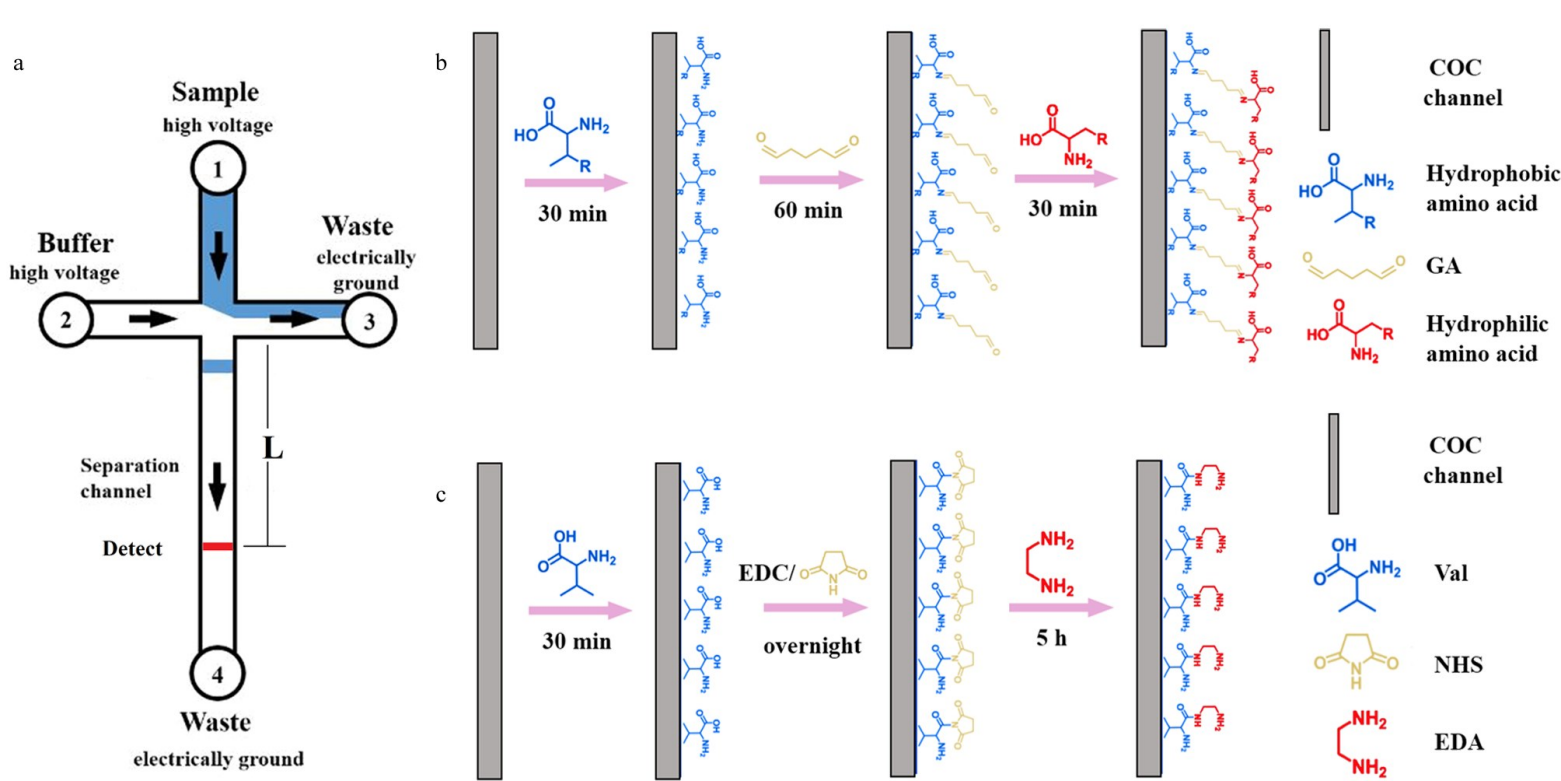
(a)芯片电泳、(b)负电荷涂层及(c)正电荷涂层COC表面改性过程的示意图

通过XPS表征改性COC芯片表面基团。通过接触角测量仪测量接触角研究COC表面亲水性。在具有负电荷涂层COC微通道内充满含有10 mmol/L NBD-F标记氨基酸混合物的0.1 mmol/L硼砂缓冲液(pH 9.3)并静置30 min,用去离子水冲洗5 min后检测,用倒置荧光显微镜拍摄COC微通道照片,使用Image J处理图片得到荧光强度图,通过荧光强度研究COC表面吸附情况。通过SEM表征改性前后微通道表面平整度。

#### 1.3.2 正电荷涂层

COC微通道表面正电荷涂层通过Val吸附、EDC/NHS羧基活化、EDA功能化构建,如图1c。首先,将2.0%(质量分数)Val/PBS溶液充满通道,室温静置30 min后用蒸馏水冲洗所有通道,38 ℃烘干;其次,将含有4.0%(质量分数)EDC/NHS的PBS溶液充满通道,室温静置过夜后用蒸馏水冲洗所有通道,38 ℃烘干;最后,将5.0%(质量分数)EDA/PBS溶液充满通道,室温静置5 h后用蒸馏水冲洗所有通道,38 ℃烘干制得微芯片,4 ℃储存备用。动态涂层通过将含有0.015%(质量分数)SDS和0.005%(质量分数)HPMC的0.1 mmol/L硼砂缓冲液引入COC通道自动形成。

通过XPS表征改性COC芯片表面基团,通过接触角测量仪测量接触角研究COC表面亲水性。在COC微通道内充满含有10 mmol/L NBD-F标记Asp与Tau的0.1 mmol/L硼砂缓冲液(pH 5.5),静置30 min,用去离子水冲洗5 min后检测,研究吸附性能。

### 1.4 MCE实验方法

依次用水和缓冲液冲洗微芯片通道5 min。将样品溶液置于储液池1,将缓冲液先填充到其余储液池中,采用门控进样模式将样品进样到分离通道中。然后在储液池1和2处施加高电压形成稳定样品流;随后,将储液池2处高电压切换到接地,实现样品进样,进样时间为1 s;进样完成后马上在储液池1和2处施加电压(*φ*),为分离通道提供合适电场,实现电泳分离。或在储液池3和4处施加高电压形成稳定样品流;随后把储液池3处高电压切换到接地,实现样品进样,进样时间1 s;进样完成后马上在储液池3和4处施加高电压,实现电泳分离。

通过LIF检测器^[24]^检测样品,通过MATLAB和Origin数据分析工具分析得到的电泳图。

### 1.5 样品制备

儿童保健品:精确称量0.02 g研磨后的儿童保健品,溶解在4.0 mL纯水中,涡旋混合1 min,超声波辅助萃取10 min,萃取液以5000 r/min转速离心10 min,上清液用0.45 μm膜过滤后,滤液待测。

运动饮料:取1 mL样品用0.45 μm膜过滤后,滤液待测。

标准品衍生化:将100 μL 25 mmol/L NBD-F甲醇溶液分别加入200 μL不同质量浓度的Lys (0.05~122 mg/L)、GABA(0.34~86 mg/L)、Tau(0.40~104 mg/L)及Asp(0.40~111 mg/L)溶液中,80 ℃水浴反应15 min,冷却至室温后,储存在4 ℃备用。

样品衍生化:将10 μL 25 mmol/L NBD-F甲醇溶液加到10 μL待测液中,衍生化方法同上。

### 1.6 液相色谱方法

保健品中Lys和GABA采用HPLC标准方法^[25,26]^做对比实验。流动相为0.05 mol/L乙酸钠水溶液-乙腈(75∶25, v/v)。采用C_18_色谱柱作为分析柱,进样体积10.0 μL,柱温为30 ℃,流速为1.0 mL/min,紫外检测波长为360 nm。

Tau和Asp采用HPLC法^[27]^做对比实验。流动相为0.05 mol/L磷酸二氢钠水溶液(质量分数为5%的氢氧化钠溶液调节pH至6.5)-乙腈(85∶15,v/v)。采用C_18_色谱柱作为分析柱,进样体积10.0 μL,柱温为40 ℃。流速为1.0 mL/min,紫外检测波长为360 nm。

## 2 结果与讨论

### 2.1 静态表面改性微芯片

#### 2.1.1 改性微芯片的性能表征

通过疏水氨基酸(以Val为例)吸附、GA固定化、亲水氨基酸(以Asp为例)功能化在COC通道表面构建Val-GA-Asp 3层静态涂层。通过XPS表征确定其是否成功涂覆在COC表面,并考察了静态涂层改性微芯片表面亲水性和抗吸附性能。

##### 2.1.1.1 涂层表征

为确定Val-GA-Asp静态涂层改性COC微芯片表面的元素组成,进行了XPS表征。如图2a, C 1*s*、N 1*s*和O 1*s*的特征峰分别出现在284.8、400.6和532.2 eV处。根据其化学结构,C 1*s*峰被拟合为3个成分。如图2b, 284.8、285.8和288.0 eV处主要成分分别来自碳碳单键(C-C)、碳氮单键(C-N)以及羧基(O-C=O)中的碳^[28]^, N 1*s*峰被拟合为1个成分。如图2c, 401.8 eV处主要成分来自C-N=C中的氮^[29]^。如图2d, O 1*s*峰被拟合为2个成分。其中531.8 eV和532.8 eV处主要成分分别来自羧基中碳氧单键(C-O)和碳氧双键(C=O)中的氧^[28]^。除了COC微芯片上有的C-C键外,其余化学键均来自Val-GA-Asp涂层,XPS表征结果证明负电荷涂层成功涂覆在COC芯片上。

**图 2:**
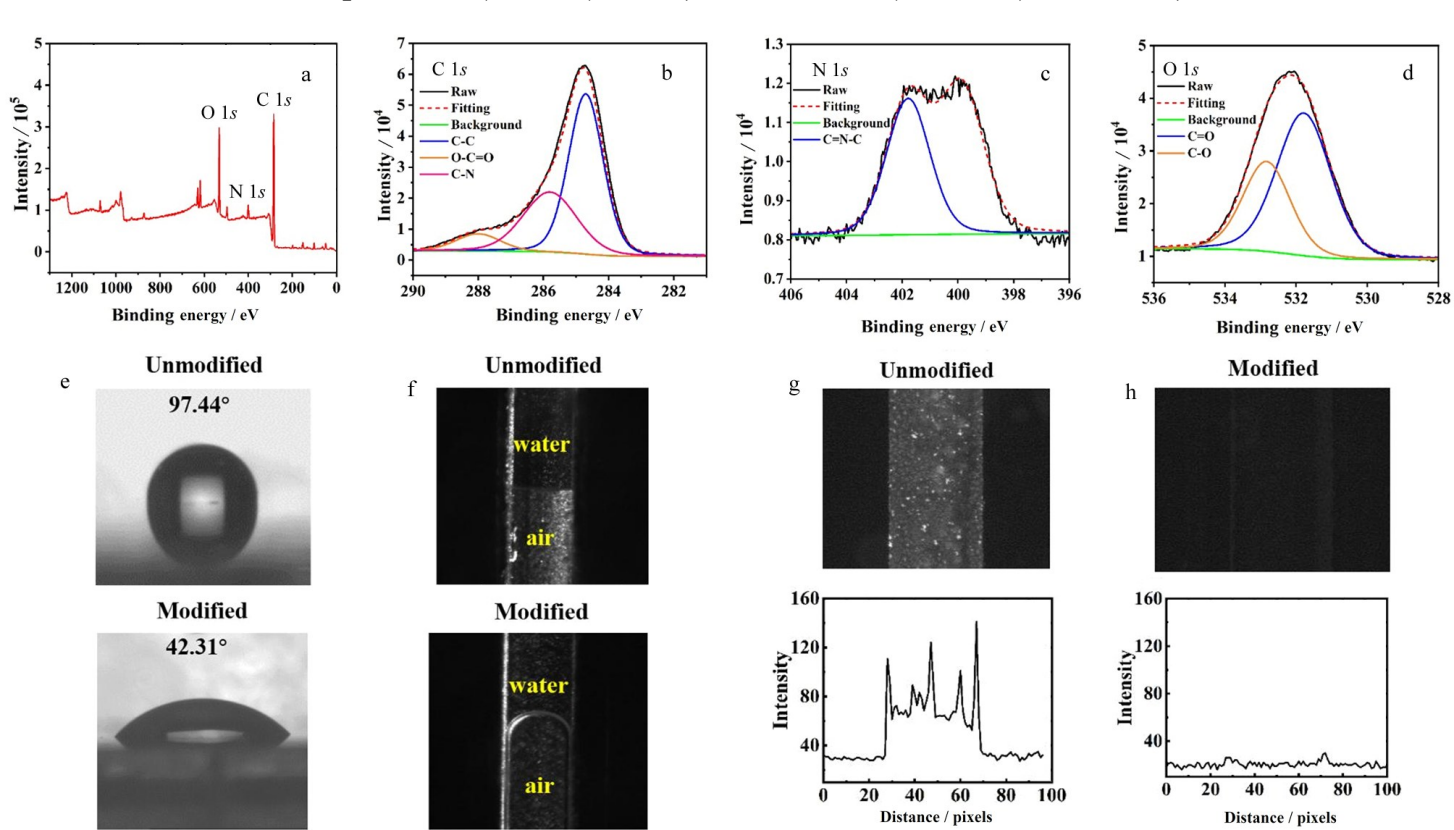
(a)静态涂层改性COC表面XPS光谱图, (b)C 1*s*、(c)N 1*s*、(d) O 1*s*峰拟合图,(e)负电荷涂层COC板上接触角,(f)COC微通道中空气-水界面轮廓图以及静态涂层(g)涂覆前和(h)涂覆后COC通道表面对氨基酸的吸附

##### 2.1.1.2 亲水性和抗吸附性能表征

采用接触角测量仪测量负电荷涂层涂覆前后COC板的水接触角,研究了COC表面亲水性。如图2e,负电荷涂层改性后,COC表面亲水性提高,水在COC表面上接触角从97.44°变为42.31°。如图2f,除了COC板上接触角减小,负电荷涂层改性后COC微通道中空气-水界面轮廓从直线变为曲线,也证明了其亲水性增强。

为研究负电荷涂层改性后COC通道表面抗氨基酸吸附能力,以0.1 mmol/L硼砂缓冲液作为背景电解质(back-ground electrolyte, BGE),将10 mmol/L NBD-F标记氨基酸混合物充满负电荷涂层改性COC微通道,去离子水冲洗后检测,荧光强度与通道内吸附量成正比,如图2g,未改性COC通道对于氨基酸有较强吸附,通道照片呈现强荧光。如图2h,负电荷涂层可有效避免通道对氨基酸非特异性吸附,改性COC通道内外荧光强度相同。

#### 2.1.2 芯片改性用氨基酸的选择

由于氨基酸侧链基团不同,负电荷涂层中第1层涂覆的疏水氨基酸涂层与第3层涂覆的亲水氨基酸涂层可能影响分析物柱效,选用带有最多正电荷的Arg作为模型分析物,研究了不同氨基酸涂层对其柱效的影响。

疏水氨基酸种类可能影响涂覆均匀性,从而影响抗吸附效果和电泳性能。将Trp、Leu、Phe、Met、Ile、Val、Ala和Pro 8种疏水氨基酸分别吸附在COC表面作为静态涂层第1层,通过GA固定化和Lys功能化构建负电荷涂层。以0.1 mmol/L硼砂缓冲液作为BGE,将0.10 mmol/L NBD-F标记Arg进样,以800 V为分离电压,得到不同涂层下的Arg电泳与理论塔板高度(*H*)图。结果表明,以Val为第1层涂层时,Arg的峰形最好,峰宽最窄(如图3a), Arg的*H*值有最小值(如图3b),即其涂层均匀性和电泳性能最好,因此选择Val为第1层疏水氨基酸涂层用于后续实验。

**图 3:**
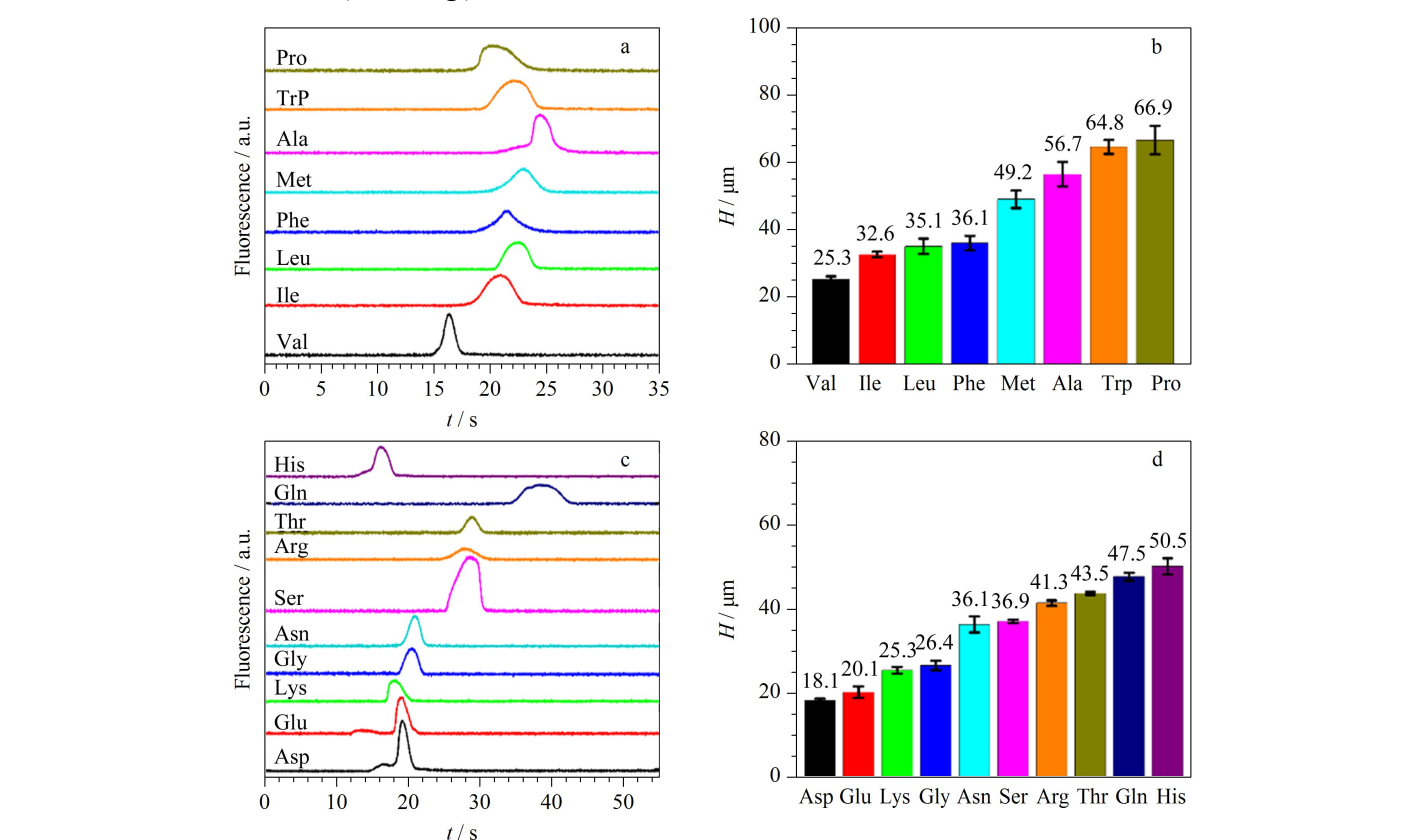
疏水氨基酸种类对(a)Arg峰形及(b)*H*的影响,亲水氨基酸种类对(c)Arg峰形及(d)*H*的影响

为得到好的分离效果,考察了第3层亲水氨基酸种类对Arg峰形与柱效的影响。通过Val吸附、GA固定化后,采用不同的亲水氨基酸作为第3层构建负电荷涂层。以0.1 mmol/L硼砂缓冲液作为BGE,将0.10 mmol/L NBD-F标记Arg进样,以800 V为分离电压,得到不同涂层下Arg电泳与*H*图。结果表明,在侧链基团含羧基的Asp作为改性第3层涂层时,Arg获得最好的峰形,峰宽最窄(如图3c), *H*最低(如图3d),柱效好。因此选择Asp为第3层亲水氨基酸涂层用于后续实验。

#### 2.1.3 电泳分离条件考察

样品移动速度(*u*) 考察了不同分离电压对Val-GA-Asp涂层改性通道的COC微芯片中Lys和GABA移动速度的影响。将0.10 mmol/L NBD-F标记Lys和0.50 mmol/L NBD-F标记GABA分别进样,在进样口下游1.5 cm处检测。如图4a, Lys和GABA的*u*均随分离电压线性增加。在同一分离电压下,Lys的*u*比GABA大,且Δ*u*随着电压增大而增大,表明负电荷涂层改性微芯片同步MCE分离分析Lys与GABA有可能性。

**图 4:**
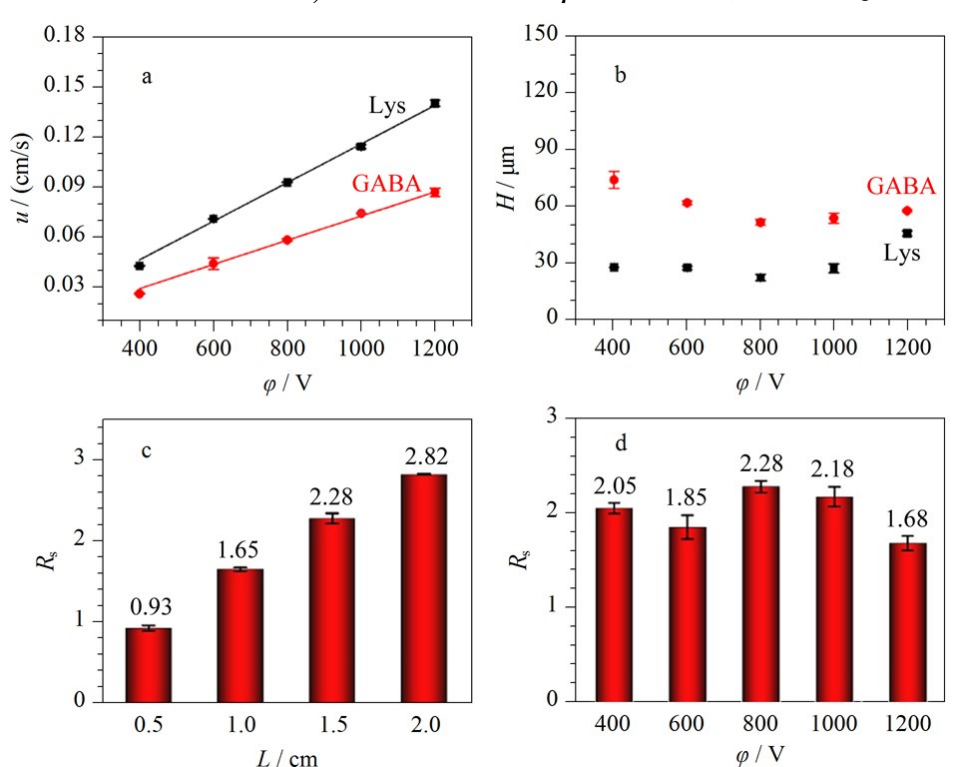
分离电压*φ*对(a)*u*以及(b)*H*的影响,(c)不同 分离距离和(d)不同分离电压下Lys和GABA的分离度(*n*=3)

柱效 用Lys和GABA的*H*与*φ*的关系表征MCE的分离性能。图4b为*φ*对COC通道中Lys和GABA迁移的影响。随着*φ*增大,Lys和GABA的*H*值先降低后增加,在800 V处取得了最小值。这符合电泳规律,能通过调节*φ*达到最佳柱效。

分离度(*R*_s_) 将Lys和GABA的混合物进样,在分离通道中检测并计算电泳图中的峰分离度,研究改性芯片的MCE分离性能及分离距离(*L*)对分离性能的影响。以0.1 mmol/L硼砂缓冲液作为BGE,将0.10 mmol/L NBD-F标记Lys和0.50 mmol/L NBD-F标记GABA混合物进样,分离电压为800 V,在分离通道不同分离距离处检测,获得一系列电泳图。如图4c, Lys和GABA的*R*_s_与*L*成正比,在*L*=2.0 cm时获得*L*最大值2.82。分离电压对分离性能的影响如图4d, Lys和GABA在*φ*=800 V时获得最大*R*_s_值2.28。因此,选择*L*=2.0 cm与*φ*=800 V用于后续实验。在此条件下,每次分离只需40 s即可完成。

#### 2.1.4 MCE分析Lys和GABA的方法学考察

在800 V分离电压下,以0.1 mmol/L硼砂缓冲液作为BGE,将NBD-F标记Lys和GABA进样,并在分离通道距离进样口2.0 cm处检测,建立了Lys和GABA的分析方法。结果表明,在0.10~122 mg/L(对应样品中含量为0.10~122 mg/kg)范围内,NBD-F标记Lys的峰面积(*A*)线性增加,*A*与分析物含量(*c*, mg/kg,下同)之间的线性方程为*A*=5.79×10^-3^*c*+2.79×10^-4^,相关系数(*r*)为0.9990; GABA线性方程在1.03~68.7 mg/L(对应样品中含量为1.03~68.7 mg/kg)范围内为*A*=1.54×10^-3^*c*+6.23×10^-4^,*r*为0.9976。Lys和GABA的检出限(LOD)分别为0.03和0.12 mg/kg (*S/N*=3);测得含量的相对标准偏差(RSD)均小于7.3%(*n*=5)。

将静态涂层改性COC微芯片的MCE-LIF方法用于儿童保健品中Lys和GABA的分析。加标前后儿童保健品的电泳图如图5, Lys和GABA的测得含量和回收率列于表1中。在儿童保健品中,检测到3.08 mg/kg Lys和2.53 mg/kg GABA, Lys和GABA的回收率分别为104%~118%和84.8%~114%, RSD≤7.2%(*n*=3)。

**表 1:** 儿童保健品中Lys和GABA的分析(*n*=3)

Analyte	This method					Original with HPLC method/(mg/kg)	Relative error/%
	Original/(mg/kg)	Spiked/(mg/kg)	Found/(mg/kg)	Recovery/%	RSD/%		
Lys	3.08±0.03	3.00	6.51	107	3.6	3.05±0.02	0.98
		6.00	10.8	118	2.1		
		12.0	15.7	104	1.0		
GABA	2.53±0.15	3.00	4.69	84.8	7.2	2.32±0.04	9.1
		6.00	8.25	96.7	7.2		
		12.0	16.6	114	2.6		

**图 5:**
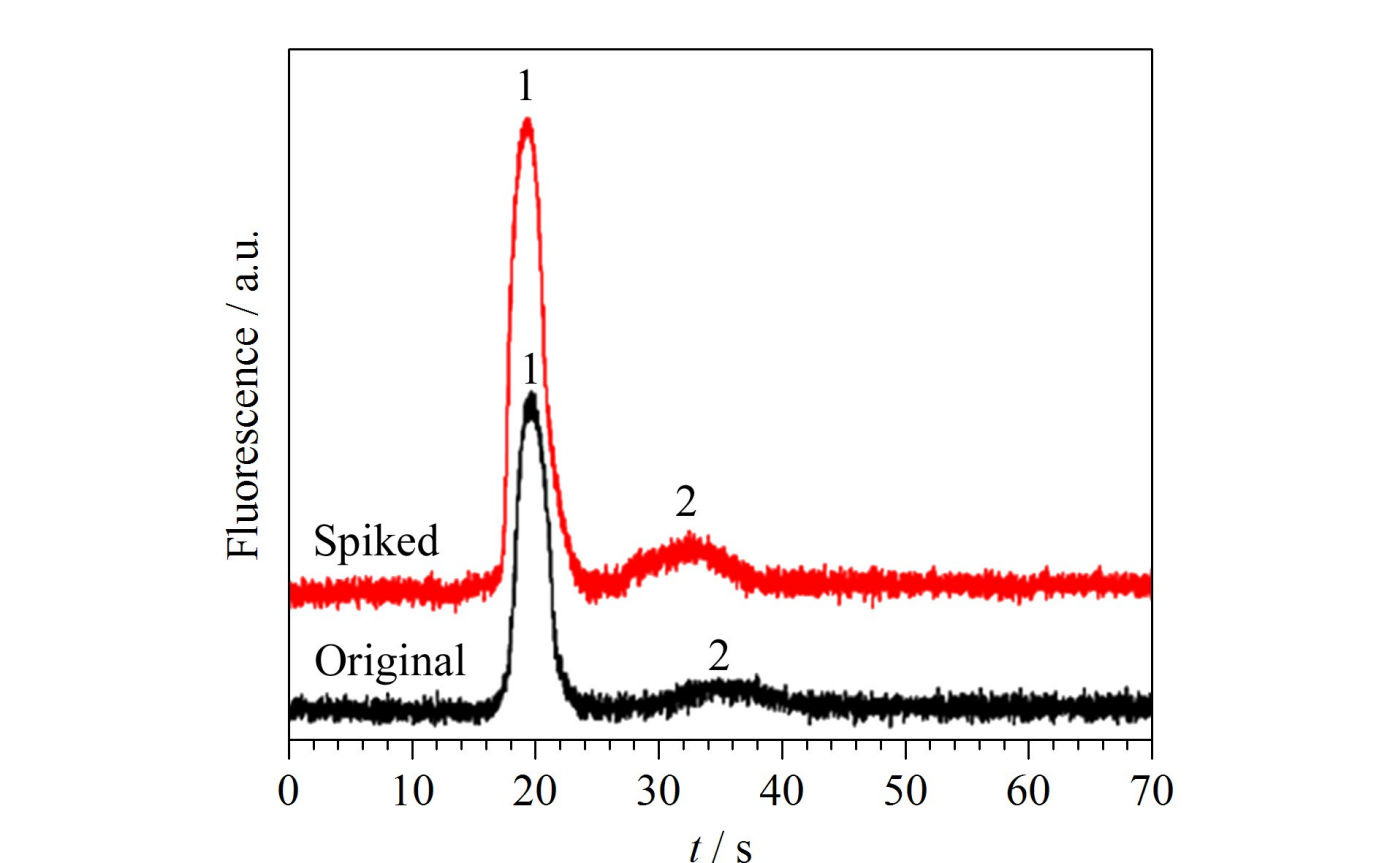
儿童保健品及3.00 mg/kg加标样品的电泳图

作为参考,儿童保健品也通过HPLC标准法进行了分析,在0.12~12.2 mg/kg范围内,Lys的线性方程为*A*=7.77×10^3^*c*-83.1,*r*为0.9993; GABA的线性方程在0.09~8.59 mg/kg范围内为*A*=3.13×10^4^*c*-1.17×10^4^,*r*为0.9987 (*n*=3)。

由表1可以看出,MCE方法测定结果与HPLC方法测定结果的相对误差分别为0.98%和9.1%。说明该方法对保健品中Lys和GABA的分离分析准确可靠。

### 2.2 动/静态结合表面改性微芯片

#### 2.2.1 改性微芯片的性能表征

根据1.3.2节表面改性方法,通过Val吸附、EDC/NHS羧基活化、EDA功能化在COC通道表面构建Val-EDA静态涂层。通过XPS表征确定静态涂层是否成功涂覆在COC微芯片表面,并考察了改性微芯片的亲水性和抗吸附性能。

涂层表征 为确定Val-EDA静态涂层改性COC微芯片表面的元素组成与表面化学特性,进行XPS表征,如图6a, C 1*s*、N 1*s*和O 1*s*特征峰分别出现在289.9、406.4和537.3 eV处。根据其化学结构,C 1*s*峰被拟合为2个成分,如图6b, 284.8和285.8 eV处主要成分分别来自碳碳单键(C-C)和碳氮单键(C-N)中的碳^[28]^。N 1*s*峰被拟合为2个成分,如图6c, 399.6 eV和400.5 eV处主要成分分别来自氨基(NH_2_)和碳氮单键(C-NH-C)中的氮^[28]^。如图6d, O 1*s*峰被拟合为1个成分,在532.2 eV处主要成分来自碳氧双键(C=O)中的氧^[28,30]^。除了COC微芯片上有的C-C键外,其余化学键均来自Val-EDA涂层,证明正电荷涂层成功涂覆在COC芯片上。

**图 6:**
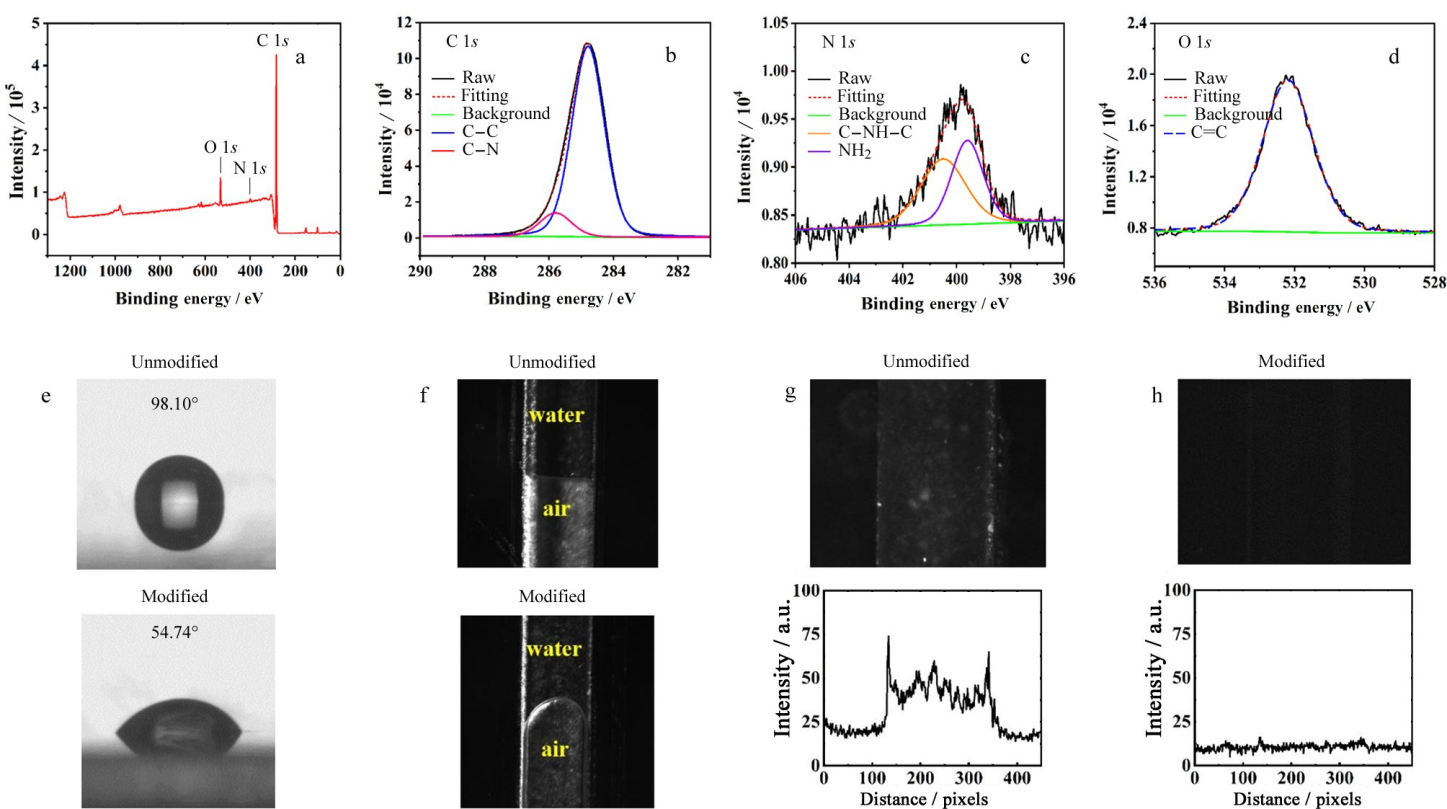
静态涂层改性COC表面(a)XPS光谱图和(b)C 1*s*、(c)N 1*s*、(d)O 1*s*峰拟合图,(e)正电荷涂层COC板上接触角和(f)COC微通道中空气-水界面轮廓,静电涂层(g)涂覆前和(h)涂覆后COC通道表面对Asp和Tau的吸附

亲水性和抗吸附性能表征 如图6e,正电荷涂层改性后,COC表面亲水性提高,水在COC表面上接触角从98.10°降到54.74°。如图6f,除了COC板上接触角减小,正电荷涂层改性后COC微通道中空气-水界面轮廓从直线变为曲线,也证明了其亲水性增强。为研究正电荷涂层改性COC微芯片通道表面抗吸附能力,以0.1 mmol/L硼砂缓冲液作为BGE,将10 mmol/L NBD-F标记Asp和Tau混合物充满正电荷涂层改性COC微通道,荧光强度与通道内目标物吸附量成正比,如图6g,未改性COC通道由于对Asp和Tau有较强吸附,通道照片呈现强荧光。如图6h,正电荷涂层可有效避免通道对Asp和Tau非特异性吸附,改性COC通道内外荧光强度相同。

#### 2.2.2 电泳分离条件考察

动态涂层改性剂浓度影响 为提高Asp和Tau的分离度,使用不带电荷的HPMC与带负电荷的SDS混合体系作为动态涂层,考察了HPMC与SDS质量分数对Asp和Tau的峰展宽(*σ*)的平方(*σ*^2^)与*u*的影响。以含有不同质量分数HPMC与SDS的0.1 mmol/L硼砂缓冲液作为BGE,将0.10 mmol/L NBD-F标记Asp和0.05 mmol/L NBD-F标记Tau混合物进样并检测。HPMC的质量分数为0.005%~0.015%, SDS的质量分数为0.005%~0.020%,硼砂浓度固定为0.1 mmol/L。如图7a, Asp的*u*随着HPMC与SDS的质量分数增加而降低,如图7b, Tau的*u*随着SDS质量分数增加先降低后增加,在0.015% SDS处取得最小值。当SDS质量分数固定为0.015%时,Tau的*u*随HPMC质量分数增加无明显变化。如图7c与7d, 当HPMC的质量分数固定为0.005%时,Asp与Tau的*σ*^2^随SDS质量分数增加先减小后增大,在0.015% SDS处取得最小值。当SDS的质量分数固定为0.015%时,Asp的*σ*^2^随着HPMC的质量分数增加而增加,而Tau的*σ*^2^随HPMC含量增加先增加后降低,在0.005% HPMC处取得最小值。在0.005% HPMC和0.015% SDS混合体系处有最小的*σ*^2^。为得到最优分离度,将0.005% HPMC以及0.015% SDS混合体系用于后续实验。

**图 7:**
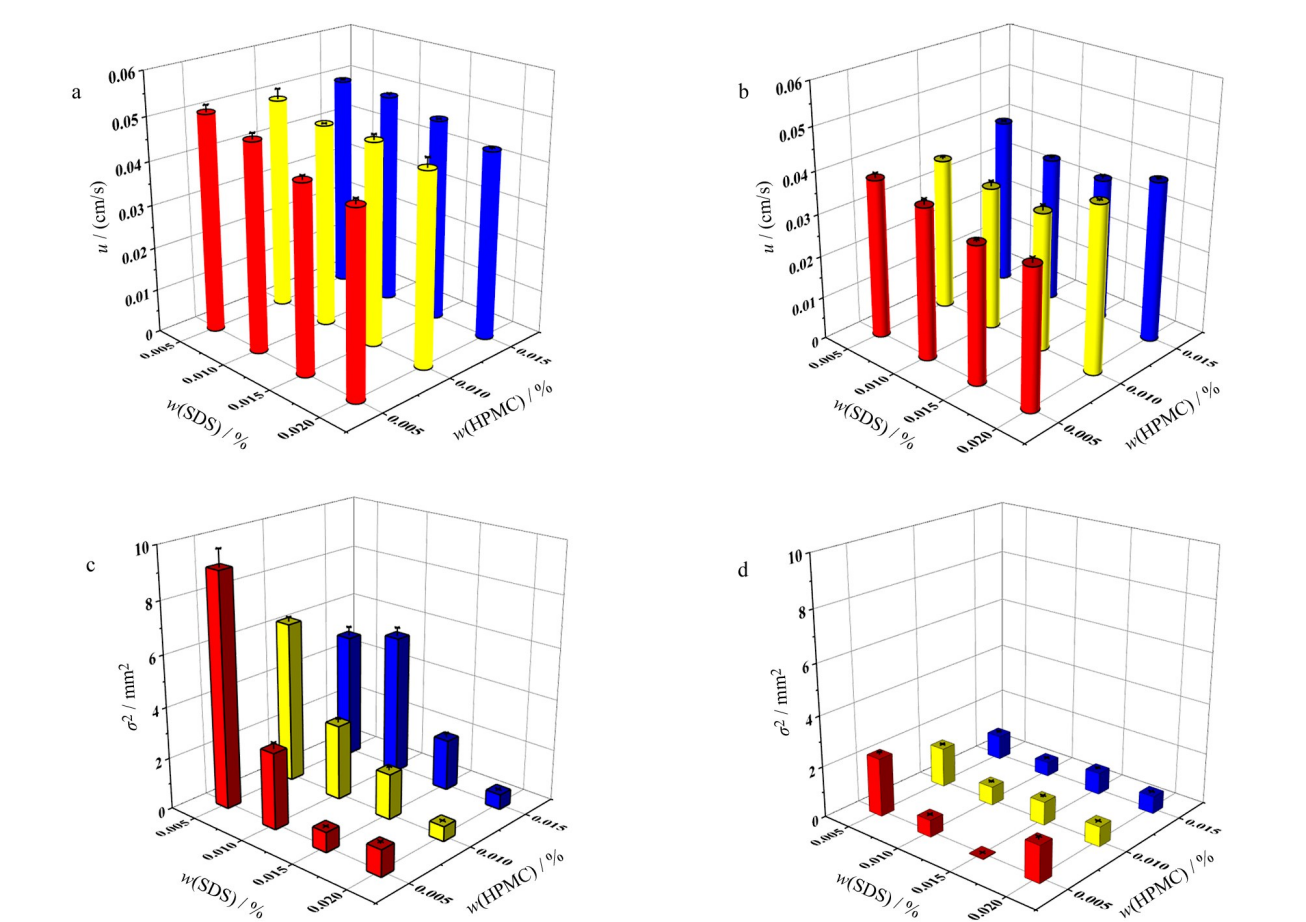
HPMC与SDS的质量分数对(a)Asp与(b)Tau *u*的影响,HPMC与SDS的质量分数对(c)Asp与(d)Tau *σ*^2^的影响(*n*=3)

样品移动速度 以含有0.005% HPMC与0.015% SDS混合体系的0.1 mmol/L硼砂缓冲液作为BGE,将0.10 mmol/L NBD-F标记Asp和0.05 mmol/L NBD-F标记Tau分别进样到仅动态涂层、仅静态涂层以及动/静态涂层结合的分离通道中,采用LIF检测。如图8a,在涂覆有各种表面涂层的分离通道中,Asp与Tau的*u*均随分离电压线性增加。在同一分离电压下,仅正电荷静态涂层通道中Asp的*u*最大,仅动态涂层COC通道中Asp的*u*最小,具有动/静态涂层结合的通道中Asp的*u*居中。如图8b, Tau的*u*受各种通道表面涂层模式的影响与Asp趋势类似。在相同条件下,Asp的*u*比Tau的大,表明改性微芯片对Asp和Tau进行MCE分离分析有可能性。

**图 8:**
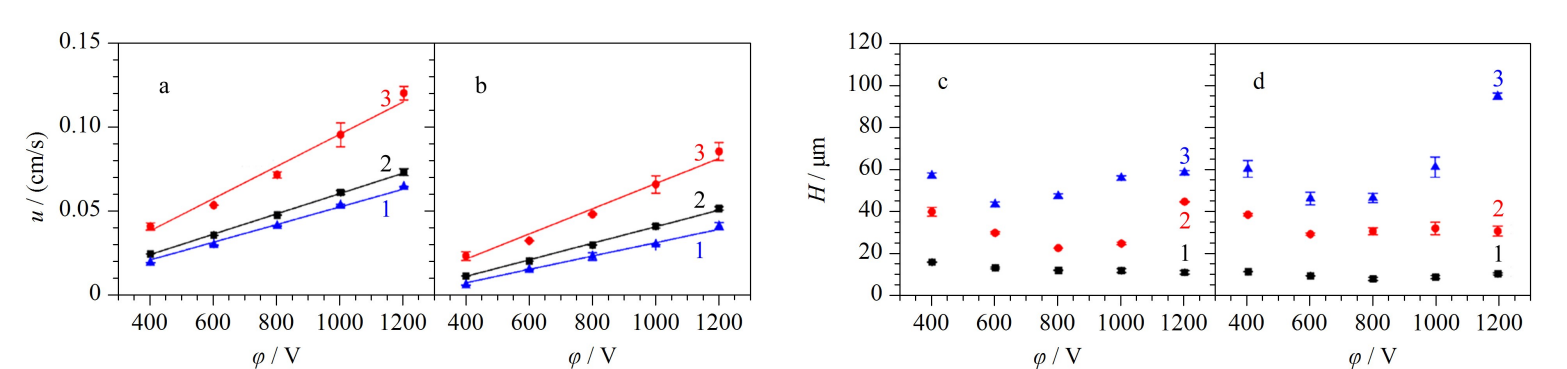
*φ*对(a)Asp和(b)Tau *u*的影响及*φ*对(c)Asp和(d)Tau *H*的影响(*n*=3)

柱效 静态涂层与动态涂层对柱效的影响会显著影响MCE分离性能。用Asp和Tau的*H*与*φ*的关系表征MCE性能。*φ*对具有不同表面涂层COC通道中Asp的影响如图8c,在所有分离电压下,随*φ*增大,Asp的*H*值先降低后增加,仅动态涂层中Asp的*H*值最大,而动/静态涂层中*H*值最小。如图8d, Tau的*H*受各种通道表面涂层模式的影响与Asp类似。动态涂层与静态涂层均对MCE柱效提高有影响,动/静态涂层结合改性通道的COC微芯片有最佳柱效。

分离度 将BGE中0.10 mmol/L NBD-F标记Asp和0.05 mmol/L NBD-F标记Tau混合物引入具有不同涂层模式的COC微通道中,以800 V为分离电压,考察不同涂层模式对Asp和Tau分离度的影响。如图9a,仅动态涂层的芯片中Asp和Tau的流速较慢但峰展宽较大,有一定的分离效果。在仅静态涂层的芯片中Asp和Tau的流速较快,有一定的分离现象。在相同条件下,动/静态涂层结合芯片加快了Asp和Tau的移动速度,减小了峰展宽,实现了Asp和Tau的完全分离,有最好的分离性能。将动/静态涂层结合改性微芯片用于后续分析,将Asp和Tau混合物进样,研究分离距离与电场强度对Asp和Tau分离度的影响。以含有0.005%(质量分数)HPMC以及0.015%(质量分数)SDS的0.1 mmol/L硼砂缓冲液作为BGE,将0.10 mmol/L NBD-F标记Asp和0.05 mmol/L NBD-F标记Tau混合物进样,分离电压设置为800 V,在不同分离距离处检测。随着分离距离增加,Asp和Tau的*R*_s_先增加后降低,在1.5 cm处取得*R*_s_最大值(如图9b)。研究了电场强度对*R*_s_的影响。如图9c, Asp和Tau在800 V分离电压下获得了最大*R*_s_值2.59。将1.5 cm分离距离与800 V分离电压用于后续分析,在此优化条件下,每次分离只需60 s即可完成。

**图 9:**
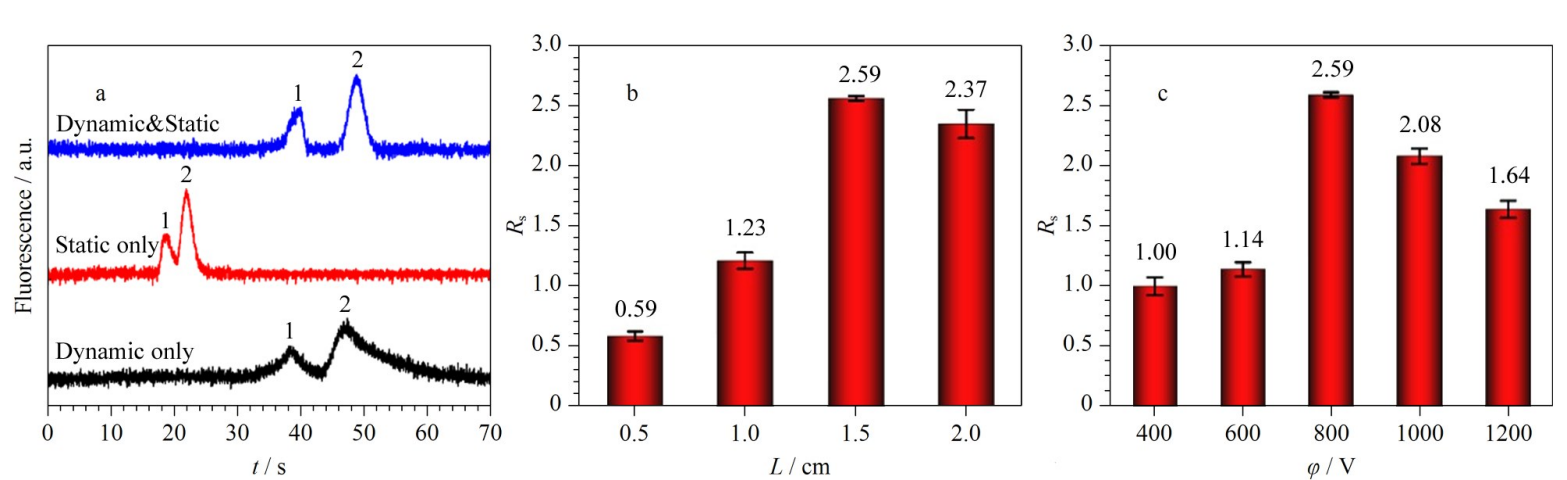
(a)涂层类型对Asp和Tau分离的影响和(b)不同分离距离、(c)不同分离电压下测得的Asp和Tau的分离度

#### 2.2.3 MCE分析Asp和Tau的方法学考察

在800 V分离电压下,以含有0.005%(质量分数)HPMC和0.015%(质量分数)SDS的0.1 mmol/L硼砂缓冲液作为BGE,将NBD-F标记Asp和Tau进样,建立Asp和Tau分析方法。结果表明,在1.33~111 mg/L(对应样品中含量为1.33~111 mg/kg)范围内,NBD-F标记Asp的峰面积线性增加,线性方程为*A*=7.25×10^-5^*c*+5.38×10^-4^,*r*为0.9968; Tau的线性方程在0.42~20.9 mg/kg范围内为*A*=7.39×10^-4^*c*+5.48×10^-4^,*r*为0.9986;在20.9~104 mg/L(对应样品中含量20.9~104 mg/kg)范围为*A*=6.28×10^-4^*c*+3.09×10^-3^,*r*为0.9989。Asp和Tau的LOD分别为0.35和0.09 mg/kg, 测得含量的RSD均小于5.6% (*n*=5)。

将具有动/静态涂层结合改性通道的MCE-LIF方法用于运动饮料中Asp和Tau的分析。加标前后运动饮料电泳图如图10。Asp和Tau测得含量和回收率列于表2中。在运动饮料中,检测到(9.89±0.55) mg/kg Asp和(15.9±0.8) mg/kg Tau, Asp和Tau的回收率分别为97.5%~105%和101%~118%, RSD≤6.4%(*n*=3)。作为参考,通过HPLC方法分析运动饮料,在0.13~13.3 mg/kg范围内,Asp的线性方程为*A*=1.41×10^4^*c*-2.03×10^2^,*r*为0.9996; Tau的线性方程在0.12~12.2 mg/kg范围内为*A*=8.12×10^3^*c*+3.06×10^2^,*r*为0.9999。表2中,MCE方法与HPLC方法测定结果的相对误差分别为1.4%和9.4%。表明该方法在保健品天冬氨酸和牛磺酸分离分析中准确可靠。

**图 10:**
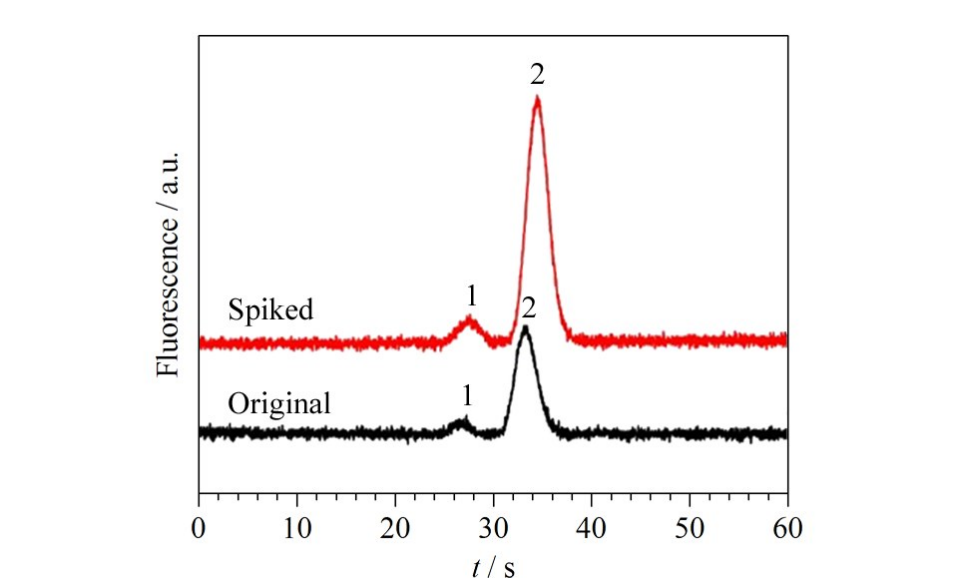
运动饮料及8.00 mg/kg加标样品的电泳图

**表 2:** 运动饮料中Asp和Tau的分析(*n*=3)

Analyte	This method					Original with HPLC method/(mg/kg)	Relative error/%
	Original/(mg/kg)	Spiked/(mg/kg)	Found/(mg/kg)	Recovery/%	RSD/%		
Asp	9.89±0.55	8.00	18.2	102	1.0	9.75±0.15	1.4
		16.0	25.3	97.5	6.4		
		32.0	43.9	105	3.6		
Tau	15.9±0.8	8.00	24.2	101	4.1	14.6±0. 1	9.4
		16.0	37.4	117	2.6		
		32.0	56.6	118	3.0		

## 3 结论

本文建立了表面改性微芯片电泳分离检测保健品中功效成分的方法,采用负电荷涂层的COC通道实现了Lys和GABA的分离,*R*_s_为2.82,正电荷涂层的COC通道实现了Asp与Tau的分离,*R*_s_为2.59。将该方法用于保健品中氨基酸的测定,回收率为84.8%~118%, RSD≤7.2%。分析结果与HPLC方法测定结果一致,该方法具有良好的应用前景,适用于儿童保健品中Lys和GABA以及运动饮料中Asp和Tau的检测。
